# Echocardiographic Correlates of Left Atrial Function Index Among Hypertensive Heart Failure Patients: A Cross-Sectional Study

**DOI:** 10.7759/cureus.38013

**Published:** 2023-04-23

**Authors:** Anthony G Kweki, Henry O Aiwuyo, Ejiroghene M Umuerri, Roy Aghwana, Oluwaseye M Oladimeji, Ugoeze N Iloeje, Fredrick I Aigbe, Austine Obasohan

**Affiliations:** 1 Internal Medicine/Cardiology, Colchester Hospital, Colchester, GBR; 2 Internal Medicine, Brookdale University Hospital Medical Center, Brooklyn, USA; 3 Medicine, Delta State University, Abraka, NGA; 4 Internal Medicine/Cardiology, Delta State University Teaching Hospital, Oghara, NGA; 5 Internal Medicine, Colchester General Hospital, Colchester, GBR; 6 Medicine, Lagos State University Teaching Hospital Hospital, Lagos, NGA; 7 Internal Medicine/Cardiology, Federal Medical Centre, Yenagoa, NGA; 8 Internal Medicine/Cardiology, Delta State University, Abraka, NGA; 9 Medicine, College of Medical Sciences, University of Benin, Benin City, NGA

**Keywords:** hypertensive heart failure, left atrial function index, correlates, sub-saharan africa, e/e’, lvef, hypertensive hf, la function index

## Abstract

Background

Heart failure contributes to the global burden of cardiovascular diseases, with hypertensive heart failure affecting individuals in their productive age group and leading to high economic losses and disability-adjusted life years. The left atrium, on the other hand, contributes significantly to left ventricular filling in heart failure patients, and the left atrial function index is an excellent tool for assessing left atrial function among heart failure patients. The study aimed to evaluate some parameters of systolic and diastolic function as correlates and potential predictors of the left atrial function index among hypertensive heart failure cohorts.

Materials and methods

The study was conducted at Delta State University Teaching Hospital, Oghara. Eighty (80) patients with hypertensive heart failure who met the inclusion criteria were enrolled in the cardiology outpatient clinics. The left atrial function index was calculated using the following formula: LAFI = (LAEF x LVOT-VTI)/LAESVI. (LAFI = left atrial function index; LAEF = left atrial emptying fraction; LAESVI = left atrial end-systolic volume index; LVOTVTI = outflow tract velocity time integral). The data were analysed using IBM Statistical Product and Service Solution Version 22. Relationships between variables were determined using analysis of variance, Pearson correlation, and multiple linear regressions. Significance was assessed at p<0.05.

Result

It was discovered that the left atrial function index correlated with ejection fraction (r = 0.616, p = 0.001), fractional shortening (r = 0.462, p = 0.001), and the ratio of early transmitral flow to early myocardial contractility, E/E' (r = -0.522, p = 0.001). However, there was no correlation with stroke volume (r = 0.38, p = 0.11); the ratio of early transmitral flow to late transmitral flow, E/A (r = -0.10, p = 0.11); isovolumetric relaxation time, IVRT (r = -0.171, p = 0.11); and tricuspid annular plane systolic excursion, TAPSE (r = 0.185, p = 0.10). Of the variables that correlated with left atrial function index, left ventricular ejection fraction and the ratio of early transmitral flow to early myocardial contractility (E/E') were found to be independent predictors of left atrial function index.

Conclusion

Left ventricular ejection fraction and the ratio of early transmitral flow to early myocardial contractility reflect changes in the left atrial function index, and as such, they should be used as surrogates for its assessment, especially in low- and medium-income countries where left atrial function index estimation is not routinely done.

## Introduction

Heart failure is a global pandemic affecting at least 26 million people worldwide, and its prevalence is on the increase, with reports showing a three-year mortality rate of patients with advanced heart failure of 57.9%-67.1% in Sub-Saharan Africa [1, 2]. In addition, hypertensive heart failure affects individuals in their productive age group with attendant high economic loss and disability-adjusted life years, and this has resulted in a loss of manpower and an increased financial burden, especially on carers [3, 4, 5].

The left atrium contributes significantly to cardiac function, even in failure, and if the left atrial mechanical and electrical function becomes impaired, cardiac output will be significantly affected [6]. Contrarily, the left atrial function index assesses the global function of the left atrium and is an excellent biomarker of left atrial function [7,8]. The left atrial function index is a composite parameter that integrates the amount of blood that enters the left ventricle during early diastole, stroke volume, and the left atrial volume index. The index is based on the assumption that reduced left atrial function will be associated with smaller cardiac output, poorer left atrial reservoir function, and larger left atrial size. In the normal subject, the left atrium maintains a normal size during normal cardiac output and contributes to that output by its ability to transfer 60%-75% of its contents to the left ventricle in early diastole. Thus, a healthy left atrium maintains a left atrial end-systolic volume index of less than 23 ml/m^2^ while functioning in the setting of normal cardiac output, which is a left ventricular output tract velocity time integral greater than 20cm [9].

In low- and medium-income countries where the left atrial function index is not routinely done [10], it is essential to assess possible parameters that may correlate with it and further find out if there are independent predictors. Therefore, the study aimed to evaluate some parameters of systolic and diastolic function as correlates and potential predictors of the left atrial function index among hypertensive heart failure cohorts.

## Materials and methods

The study was conducted at the Delta State University Teaching Hospital, Delta State, Nigeria. The study period was between June 2020 and October 2021. The study population included eighty (80) hypertensive heart failure patients who enrolled in the heart failure follow-up clinic. The study design was cross-sectional. Hypertensive heart failure patients aged 18 years and older who gave consent were included in the study. Those with subclinical echocardiographic views, atrial fibrillation, and atrial flutter who refused or lacked the capacity to grant consent, as well as those with comorbidities such as chronic obstructive pulmonary disease, valvular heart disease, diabetic mellitus, retroviral disease, autoimmune disorders, and chronic kidney disease (estimated glomerular filtration rate (eGFR) < 60mls/min/1.73m^2^) were excluded from the work. Hypertensive heart failure was defined as heart failure patients who have a clinical history of hypertension, irrespective of their current blood pressure status, or persistent BP ≥ 140/90 mmHg in those who are not previously known to be hypertensive and/or on antihypertensive medications [11, 12]. A semi-structured interviewer-administered questionnaire was used to collect information from hypertensive heart failure individuals attending the heart failure clinic, which included age, sex, occupation, and echocardiographic parameters.

To obtain data from the heart failure patients, a transthoracic echocardiogram (Xario Diagnostics ultrasound system model SSA-660A, Toshiba Medicals, probe frequency of 3.5 MHz) was used alongside electrocardiographic gating by the researcher at the time of recruiting patients. The left atrial function was determined by the volumetric method using the left atrial function index, as shown in the formula below. The left atrial volumes were estimated at different time points of the cardiac activity: the maximal left atrial volume was calculated at the end of the T wave on the electrocardiogram (ECG), just before the opening of the mitral valve; the minimal left atrial volume was at the beginning of the QRS complex, just at the closure of the mitral valve; and the preceding left atrial contraction volume was conducted at the beginning of the P wave [13]. The left ventricular outflow tract velocity time integral was assessed by placing the sample volume of the pulse wave Doppler in the LVOT-VTI, and the velocity time integral of spectral tracing was traced, and then the value was obtained from the machine. Based on these parameters, the left atrial function of hypertensive heart failure patients was calculated with the formula below. The left atrial emptying fraction was calculated from the difference between left atrial end-systolic volume and left atrial end-diastolic volume and divided by the LA end-systolic volume, as shown below. The left atrial function was assessed by the left atrial function index [9]: LAFI = (LAEF x LVOT-VTI)/LAESVI, where: LVOT-VTI = velocity time integral of the left ventricular outflow tract (cm); LAESV = maximal left atrial volume in end-systole (ml); LAFI = left atrial function index; LA emptying fraction = (LAESV - LAEDV)/LAESV.

The early transmittal velocity by pulsed wave Doppler was obtained with the sample volume set at the tips of the mitral leaflets. Peak velocities in early diastole (E-wave velocity during early left ventricular relaxation) and late diastole (A-wave velocity during atrial contraction) were measured, and the ratio E/A was calculated. When atrial contraction occurs before the mitral deceleration has decreased to zero, the deceleration time is estimated as the time between the peak E-wave velocity and the deceleration slope extrapolated to zero baseline. Isovolumetric relaxation time was estimated from the leading edge of the aortic valve closure point to the leading edge of the mitral valve opening point.

In order to determine the ratio of early transmitral flow to early myocardial contractility (E/E’), pulsed wave Doppler tissue imaging was used to measure peak velocities in systole (S’), early diastole (E), and late diastole (A’), with the sample volume placed at the septal annulus [14]. The E/E’ was then calculated as a measure of left ventricular end-diastolic pressure, where necessary [15].

Left ventricular systolic function was assessed by ejection fraction, fractional shortening, and stroke volume. In 2D echocardiography [15], the measurement of left ventricular diameter was taken just below or at the tip of the mitral valve leaflets in the two-chamber or four-chamber view, or the left ventricular diameter was taken exactly through the centre point of the left ventricular cavity in short axis views, either at basal or mid-papillary level, during diastole and systole. These measurements will help us calculate the ejection fraction and fractional shortening of the left ventricle in that plane in the following equation: EF = (LVIDd3-LVIDs3)/LVIDd3 100; FS = LVIDd-LVIDs/LVIDd, where LVIDd = LV internal diameter at end-diastole and LVIDs = LV internal diameter at end-systole.

M-mode echocardiography [15] was used to assess left ventricular dimensions. The M-mode cursor was placed just at the tip of the mitral leaflets, or exactly perpendicular to the inferior wall, passing through the centre of the left ventricular cavity, and this gave rise to the M-mode trace. The measurements of the left ventricular dimension were timed with electrocardiogram tracing. The measurement of the left ventricular dimension that is done when the time cursor is placed at or immediately before the peak of the R-wave in the QRS complex was considered the left ventricular internal diameter in diastole, LVIDd, and the LV dimension at the end of the T-wave in the ECG was taken for the left ventricular internal diameter in systole, LVIDs. This was applicable for all 2D and M-mode measurements. Left ventricular systolic dysfunction was considered present when the LVEF <50% [16]. Right ventricular systolic function was assessed using the tricuspid annular plane systolic excursion (TAPSE).

Data were collated and analysed with International Business Machines Statistical Product and Service Solutions (IBM-SPSS) version 22.0. A questionnaire was used to obtain information from participants. Means and standard deviation were used to present continuous variables. Pearson’s correlation test was used to assess the relationship between the left atrial function index and left ventricular function parameters such as ejection fraction, fractional shortening, stroke volume, the ratio of early transmitral flow to early myocardial contractility, tricuspid annular plane systolic excursion, etc. The variables that significantly correlated with the left atrial function index were assessed further for cause and effect with multiple linear regressions. A positive correlation was defined by a correlation coefficient of r <+1. On the other hand, a negative correlation was defined by r < -1. The strength of the correlation was assessed as follows: weak correlation: r = 0.10-0.30; moderate correlation: r = 0.40-0.60; and strong correlation: r ≥0.70. Significance was assessed at p<0.05.

Ethical approval was obtained from the Health Research and Ethics Committee of the Delta State University Teaching Hospital, Oghara, with the institutional review board number HREC/PAN/2019/0313. Patients were duly informed, and consent forms were duly signed or thumb printed before they were enrolled in the study. The researcher also maintained confidentiality and the right to exit the study at any time.

## Results

Sociodemographic characteristics of hypertensive heart failure patients

Table 1 shows the sociodemographic characteristics of the study population, with 52.5% of the hypertensive heart failure patients presenting at an age distribution of 60-69 years with a male: female ratio of 1.2:1. Thirty-five percent and 26.3% of hypertensive heart failure patients are traders and artisans, respectively, with 38.7% of them attaining a secondary school level of education.

**Table 1:** Sociodemographic characteristics of hypertensive heart failure patients

Sociodemographic variable	Hypertensive heart failure n(%)
Age (in years)	
40-49	5 (6.3)
50-59	20 (25)
60-69	42 (52.5)
70-79	11 (13.8)
≥80	2 (2.5)
Gender	
Male	44 (55)
Female	36 (45)
Occupation	
Trader	28 (35)
Farmer	13 (16.3)
Civil servant	18 (22.5)
Artisan	21 (26.3)
Education level	
No formal education	13 (16.3)
Primary	12 (15)
Secondary	31 (38.7)
Tertiary	24 (30)

Correlation between left atrial function index and left ventricular systolic function

Table 2 shows a correlation between left atrial function and left ventricular systolic function. Left atrial function correlated moderately with ejection fraction (r = 0.616, p = 0.001) and fractional shortening (r = 0.462, p = 0.001). There is no correlation between the left atrial function index and stroke volume (r = 0.184, p = 0.11).

**Table 2:** Correlation between left atrial function and left ventricular systolic function among hypertensive heart failure patients LAFI: left atrial function index; LVEF: left ventricular ejection fraction; FS: fractional shortening; SV: stroke volume; *significant at p 0.05; **compared using Pearson’s correlation

LV systolic function	LAFI	
	Correlation coefficient (r)**	p-value
LVEF (%)	0.616	0.001*
FS (%)	0.462	0.001*
SV (ml)	0.184	0.11

Correlation between left atrial function index and left ventricular diastolic function

Table 3 shows the correlation between the left atrial function index and the left ventricular diastolic function. The left atrial function index correlates moderately with E/E' (r = -0.522, p = 0.001). The left atrial function index did not correlate with E/A (r = -0.10, p = 0.110) or IVRT (r = -0.171, p = 0.11). 

**Table 3:** Correlation between left atrial function and left ventricular diastolic function among hypertensive heart failure patients LAFI: left atrial function index; E/A: ratio of mitral early inflow velocity to late inflow velocity; IVRT: isovolumetric relaxation time; E/Eˈ: ratio of mitral early inflow velocity to early myocardial contraction; *significant at p<0.05, **compared using Pearson’s correlation

LV diastolic function	LAFI	p-value
	Correlation coefficient (r)**	
E/A	-0.10	0.38
E/E´	-0.522	0.001*
IVRT (sec)	-0.171	0.13

**Figure 1:**
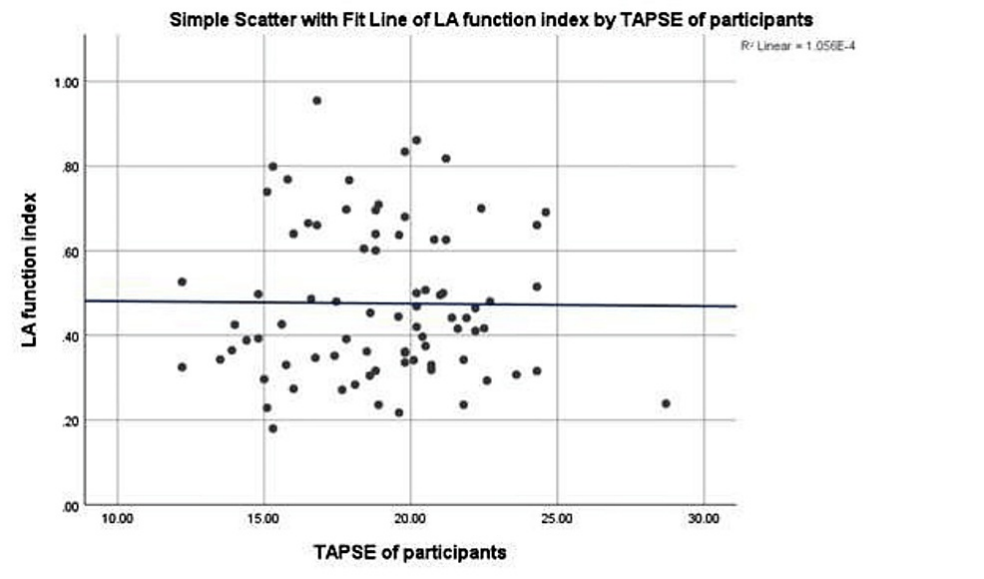
Correlation between left atrial function index and right systolic function among hypertensive heart failure patients LA: left atrial; TAPSE: tricuspid annular plane systolic excursion

Figure 1 shows the correlation between the left atrial function index and right ventricular systolic function. There is no correlation between the left atrial function index and TAPSE (r = 0.185, p = 0.10) among hypertensive heart failure patients.

Independent predictors of left atrial function index among hypertensive heart failure patients

Table 4 shows a multiple regression analysis illustrating independent predictors of the left atrial function index in hypertensive heart failure individuals. Reduced ejection fraction (Beta = 0.267, p = 0.01) and elevated E/E' (Beta = -0.285, p = 0.001) independently predict lower LAFI. On the other hand, abnormal fractional shortening (Beta = 0.03, p = 0.75) did not independently predict lower left atrial function.

**Table 4:** Multiple regression analysis showing independent predictors of LAFI among hypertensive heart failure patients Dependent variable: LAFI; LVEF: left ventricular ejection fraction; E/E’: ratio of early transmitral flow to early myocardial contractility; TAPSE: transannular plane systolic excursion; SE: standard error; *significant at p<0.05

Predictors of LAFI	Unstandardized coefficients		Standardized coefficients	t test	p-value
	B	SE	Beta		
(Constant)	16.540	11.810		1.40	0.16
LVEF (%)	0.474	0.187	0.267	2.54	0.01*
E/E'	-1.214	0.359	-0.285	-3.38	0.001*
FS (%)	0.078	0.242	0.030	0.32	0.75

## Discussion

In our study, the left atrial function index correlated moderately and positively with left ventricular ejection fraction and fractional shortening, but there was no correlation with stroke volume. This was comparable to a report from another author [17] that noted that ejection fraction significantly increases with higher values of the left atrial function index. It was also similar to a report by another author [18] that demonstrated ejection fraction to have a weak correlation with maximum left atrial volume and left atrial emptying fraction. The association between left atrial function and ejection fraction further showed that as the ejection fraction of heart failure patients began to decline, the left atrial function was also affected, and vice versa. In other words, these parameters are mirror images of each other, and as a result, the left atrial function index can act as a biomarker of the left ventricular ejection fraction and vice versa. The lack of correlation between stroke volume and the left atrial function index in this study may be due to the poor sensitivity of stroke volume in defining left ventricular systolic function as compared to the ejection fraction.

The left atrial function index was inversely correlated with the ratio of early transmitral inflow to early myocardial tissue contractility, but there was no correlation with the ratio of early transmitral flow to late transmitral flow or isovolumetric relaxation time. This finding was comparable to a report from another study in the United States [19] that showed the left atrial function index to be inversely correlated with the ratio of early transmitral inflow to early myocardial tissue contractility. Another study that was conducted in nine centres in Japan and one centre in Germany [20] also reported an inverse correlation with the ratio of early transmitral inflow to early myocardial tissue contractility. They made use of speckle-tracking echocardiography to determine the left atrial function, unlike the index study, which used a volumetric method. The relationship between the left atrial function index and the ratio of early transmitral inflow to early myocardial tissue contractility suggests that left atrial function decreases with impaired left ventricular relaxation and compliance. It also showed that the left atrial function index declines as heart failure patients develop elevated left ventricular end-diastolic pressure. Furthermore, the absence of correlation of left atrial function with the ratio of early transmitral flow to late transmitral flow and isovolumetric relaxation time may be a result of the inability of these parameters to accurately define left ventricular relaxation abnormalities and stiffness. Even though our work did not find a correlation between these variables, a Chinese study [21] reported a significant correlation between the left atrial function index and the ratio of early transmitral flow to late transmittal flow. Their study population was made up of hypertensive patients. This work was carried out among hypertensive heart failure patients.

In addition, this study also demonstrated that the left atrial function index had no correlation with tricuspid annular plane systolic excursion, and this may be due to the fact that the population of this study was mostly patients with chronic stable heart failure who were routinely followed up in the clinic. A study from Nigeria [22] reported a similar finding, but it is different from another work from the same country [23] that studied the right ventricular function in patients with heart failure in a cardiac clinic and demonstrated that left atrial diameter correlated negatively with tricuspid annular plane systolic excursion.

Although ejection fraction and the ratio of early transmitral flow to early myocardial contractility independently predict an abnormal left atrial function index, this was not the case with fractional shortening. Ejection fraction takes into consideration left ventricular myocardial contractility and left ventricular volumes, unlike fractional shortening, which considers only left ventricular myocardial contractility. This may account for the inability of fractional shortening to be an independent predictor of left atrial function among hypertensive heart failure patients.

Limitations of the study

The study was conducted in a single hospital and may not represent the true picture of the population. Also, chronic obstructive lung disease was excluded on clinical grounds, and patients with subclinical conditions may have been included; therefore, they may have had an impact on the right ventricular assessment. Transoesophageal echocardiography, speckle tracking, and cardiac magnetic resonance imaging are better tools to assess left atrial function. This was not used due to challenges with funds.

## Conclusions

There was a correlation between the left atrial function index and left ventricular ejection fraction, fractional shortening, and the ratio of early transmitral inflow to early myocardial tissue contractility, but there was no correlation between left atrial function index and stroke volume, the ratio of early transmitral inflow to late transmitral inflow, isovolumetric relaxation time, or tricuspid annular plane systolic excursion in our study. Left ventricular ejection fraction and the ratio of early transmitral inflow to early myocardial tissue contractility independently predict abnormal left atrial function index and could be a possible alternative for the left atrial function index, especially in low-and middle-income countries where its calculation is not commonly implemented.
